# Impact of Generative AI-assisted programming on the computational thinking of high school students

**DOI:** 10.3389/fpsyg.2026.1703177

**Published:** 2026-03-24

**Authors:** Rong Guo, Guian Li, Haifei Miao, Zhongling Pi, LiHua Xie

**Affiliations:** 1 Faculty of Education, Shaanxi Normal University, Xi’an, China; 2 School of Physics and Information Technology, Shaanxi Normal University, Xi’an, China; 3 Key Laboratory of Modern Teaching Technology, Shaanxi Normal University, Xi’an, China

**Keywords:** computational thinking, Generative AI, high school, mixed-methods design, text-based programming

## Abstract

**Introduction:**

Generative AI has demonstrated remarkable performance in the field of education due to its powerful text-generation capabilities. However, its impact on programming education is still in the early exploration stage, with relatively few related empirical studies.

**Methods:**

This research explored the impact of Generative AI-assisted programming on high-school students’ computational thinking. It recruited 83 high-school students. This study adopted a convergent parallel mixed-methods design. First, quantitative data was analyzed to measure changes in computational thinking. Subsequently, follow-up interviews were conducted with selected participants to explain and elaborate on the quantitative research findings.

**Results:**

The computational thinking (*p* < 0.01) of the experimental group has demonstrated a significant improvement. The analysis revealed significant, differentiated gains: in computational thinking, algorithmic thinking and abstraction improved substantially (*p* < 0.01). These quantitative outcomes were further illuminated by qualitative findings, which highlighted Generative AI’s role in providing real-time, personalized scaffolding-a key mechanism driving student cognitive skill development.

**Discussion:**

Generative AI-assisted programming learning strategy promotes personalized learning and stimulates students’ enthusiasm for learning programming. The findings suggest the potential for applying Generative AI-assisted programming in similar educational settings.

## 1 Introduction

In the past 2 years, the rise of Generative AI technology has drawn widespread attention. Represented by ChatGPT, Generative AI had hundreds of millions of users in just a few months after its release on November 30, 2022, which fully demonstrates its charm (Bai et al., 2024). With its super strong language learning ability and the ability to respond in a human like manner, Generative AI has aroused extensive interest among educators. Experts and scholars have started to explore how Generative AI can empower education. Existing research shows that Generative AI can significantly enhance students’ high order thinking skills such as critical thinking (Essien et al., 2024) and reflective thinking (Lin et al., 2025).

Computational thinking (CT), as one kind of high order thinking abilities, is an essential skill that students in the 21st century must possess (Kong, 2016). The core of computational thinking lies in cultivating students’ capabilities to solve problems, design systems, and understand human behavior by applying computer science concepts (Wing, 2006). Programming is an ideal tool for cultivating students’ computational thinking (Hsu et al., 2018) and can significantly enhance their computational thinking abilities (Aksit and Wiebe, 2020).

However, in actual teaching, students encounter significant difficulties in learning text-based programming (ÖzdiNç and Altun, 2014). Text-based programming is inherently difficult and imposes a high cognitive load. It contains a large amount of trivial grammatical knowledge and logical relationships. Students need to have a certain foundation to read and write code. However, most students have no programming background. Confronted with trivial grammar and dull code, students exhibit low self-efficacy. Therefore, in the learning of text-based programming, students need certain teaching support to assist their learning, maintain a high level of enthusiasm, and ultimately enhance their computational thinking (Bakar et al., 2020). Generative AI can explain grammar and code to students, respond quickly to their questions, and provide solutions to the above-mentioned problems.

Based on the issues outlined above, this study aims to investigate the effects of Generative AI-assisted programming on high school students’ computational thinking and its respective sub-dimensions. To this end, a convergent parallel mixed-methods design was employed, and a 12-week quasi-experimental study was conducted with 83 students from a high school.

## 2 Literature review

### 2.1 Programming learning and its impact on computational thinking

Programming education is regarded as an effective vehicle for cultivating and developing students’ computational thinking skills (Lye and Koh, 2014). This is because programming provides a pathway for applying computational concepts and practices while simultaneously supporting the development of cognitive skills associated with computational thinking, such as algorithmic thinking, abstraction, decomposition, and pattern recognition (Basogain et al., 2018). Specifically, the process of problem-solving through programming first requires eliminating irrelevant information from complex problem situations to extract the essential problem (abstraction). Second, the problem must be broken down into manageable modules (decomposition). The specific steps for solving the problem are then theoretically refined (algorithmic thinking), and common structures for solving problems across different contexts are identified (pattern recognition). Third, a program structure is formed to address the current problem (Boom et al., 2022; Shute et al., 2017). Thus, the cognitive processes involved in programming closely align with the core elements of computational thinking.

The current state of using programming to cultivate students’ computational thinking. In recent years, programming education has become increasingly prevalent in K-12 settings. Block-based programming has gained popularity due to its “low floor, high ceiling” characteristics (Witherspoon et al., 2018). Students can program simply by “dragging and dropping blocks.” Features such as ease of operation, short development cycles, and minimal prerequisite programming knowledge have made block-based programming particularly popular in lower and middle grade levels (Yang and Lin, 2024). However, some studies have noted the limitations of block-based programming in cultivating deeper computational thinking abilities (Sun and Liu, 2024). These limitations manifest as follows: the “drag-and-drop” approach may hinder students’ deep understanding of underlying abstract programming concepts, making it difficult to fully develop their computational thinking. Block-based environments often reduce difficulty through encapsulation and simplification, which may inadvertently bypass opportunities to cultivate certain advanced computational thinking skills (Grover et al., 2017). Consequently, students may not receive sufficient practice in handling complex logic or optimizing algorithm efficiency.

In contrast, text-based programming offers significant advantages in cultivating students’ computational thinking. Text-based programming promotes students’ deep understanding of abstract concepts and code logic, a process that helps them master core CT elements such as abstraction and algorithmic thinking (Sun and Liu, 2024). Conducted in an environment of writing and debugging code, text-based programming provides a more authentic problem-solving context, effectively fostering students’ problem-solving abilities and systematic modeling skills (Deng et al., 2020). The abstract and universal nature of text-based programming makes the thinking patterns cultivated within it more transferable to other domains and more advanced learning contexts. However, research on text-based programming instruction for K-12 students, particularly high school students, and its impact on CT sub-skills, remains relatively limited.

Despite the potential advantages of text-based programming for CT development, high school students face numerous challenges during the learning process. Programming involves complex syntactic rules, rigorous logical structures, extensive syntax knowledge, and the debugging skills required to handle syntactic or logical errors (Yurdakök and Kalelioğlu, 2024). Research confirms that learning programming syntax often leads to frustration for programming novices, especially young students. Additionally, students commonly report that the testing and debugging process is the most challenging aspect; when code fails to run as expected, students can easily become confused and frustrated (Leonard et al., 2021). Due to these factors, students gradually lose interest in learning programming, which seriously hinders the development of their CT skills (Sung et al., 2017). Bakar et al. (2020) noted that even minor negative experiences during the initial learning stage can discourage students, subsequently affecting their potential in programming learning. The underlying cause of these problems is the complexity of programming knowledge, which leads to cognitive overload. Faced with this challenge, students require timely and personalized support to mitigate the difficulties encountered in programming learning. However, a current paradox is that traditional lecture-based instruction often struggles to accommodate individual differences among students, failing to provide each student with timely, personalized support. In recent years, the rapid development of Generative AI has opened new pathways for personalized learning support.

### 2.2 The advantages of Generative AI in supporting programming learning

Generative AI is a technology that uses deep learning models to generate human-like content (including images and text) in response to complex and diverse prompts (e.g., language, instructions, and questions) (Lim et al., 2023). Focusing on the programming domain, the affordances of Generative AI can directly address the aforementioned difficulties in programming learning, such as a lack of syntactic knowledge, incomprehension of code meaning, and debugging challenges. Specifically, it can assist learning through forms like explaining code (Yilmaz and Karaoglan Yilmaz, 2023b), correcting code and analyzing causes, and providing rapid responses (Qureshi, 2023). More importantly, its mechanism of action can be explained from a theoretical perspective (see Table 1).

**TABLE 1:** Conceptual mechanisms of Generative AI in supporting the development of computational thinking.

Core function of Generative AI	Potential impact on programming learning	Likely enhanced CT skills	Theoretical underpinning
Dynamic scaffolding	Provides timely support, breaking down complex tasks into manageable steps.	Decomposition, algorithmic thinking	Zone of proximal development; scaffolding theory
Immediate, personalized feedback	Instant feedback helps prevent the solidification of misconceptions.	Debugging	Formative assessment
Reducing extraneous cognitive load & increasing germane cognitive load	Handles low-level tasks (e.g., syntax, functions), freeing students’ cognitive resources for higher-order thinking.	Algorithmic thinking	Cognitive load theory
The use of natural language dialogue for program debugging	Externalizes programming thought processes.	Abstraction	Social constructivism

The code explanation function is not merely about helping students understand the meaning of code; it is also a process of cognitive reallocation (Omeh et al., 2025). When handling programming tasks, students are involved in many low-level, repetitive, and memorization-heavy tasks, such as syntax and functions. These tasks consume substantial cognitive resources, creating a significant extraneous cognitive load. According to Cognitive Load Theory, the more elements processed simultaneously, the heavier the load on working memory (Sweller, 2010). When using Generative AI for programming learning, it can assist students in recalling syntax, functions, etc., thereby freeing them from tedious syntax and time-consuming debugging. Students can then focus their valuable cognitive resources on enhancing higher-order thinking skills such as exploring the nature of problems, decomposing problems, and designing algorithms. Instead of consuming cognitive resources on questions like “where does the semicolon go,” they can think about which algorithm can improve program efficiency. By explaining syntax and providing examples according to student needs, Generative AI helps reduce extraneous cognitive load and optimize germane cognitive load, enabling students to concentrate on issues like algorithm design and problem definition, thereby fostering skills such as abstraction and algorithmic thinking.

Code correction assists students in locating errors while externalizing implicit thought processes, facilitating knowledge construction. Social constructivism posits that dialogue is the externalization of thinking; students clarify, organize, and deepen their thinking through conversation. Students can discuss the reasons for program errors with Generative AI through natural language dialogue and complete debugging with its assistance. Through multiple iterations of “debugging-feedback-correction,” students construct their understanding. This process compels students to externalize their thinking, comprehend Generative AI’s output, and test it within the Python environment. To make themselves understood by Generative AI, students must organize their chaotic thoughts—a form of thinking about thinking, which represents a higher level of abstraction. Therefore, the process of conversing with Generative AI contributes to the development of students’ abstraction skills.

Timely and personalized responses initiate heuristic dialogues that help students decompose problems. By guiding students in analyzing problems and providing immediate, personalized feedback, Generative AI acts as a “more capable tool.” When students encounter programming tasks beyond their current ability, Generative AI can help them reach their “potential development level” by providing examples, hints, etc., With Generative AI’s assistance, students can complete more complex tasks than they could handle alone. They need to understand Generative AI’s prompts and then construct the next steps. This is a classic application of scaffolding within the Zone of Proximal Development. The process of transforming Generative AI’s prompts into concrete steps and planning the next move helps students develop decomposition and algorithmic thinking skills. Furthermore, this immediate, personalized feedback realizes formative assessment, where errors are pointed out as they occur, preventing the reinforcement of misconceptions. The iterative cycle of “attempt → feedback → revise → re-attempt” aids in developing debugging skills within computational thinking.

### 2.3 Computational thinking

In exploring the concepts and competencies of CT, scholars have endeavored to elucidate its core elements. Papert first introduced the concept of computational thinking, understanding it as a form of procedural thinking. However, the precise connotation of CT remains a subject of debate. In 2006, Wing (2006) defined it as using fundamental concepts of computer science to solve problems, design systems, and understand human behavior. This definition fundamentally reshaped the perception of computer science’s value, yet its abstract nature made it difficult to guide specific teaching practices. Brennan and Resnick (2012) proposed a more operational three-dimensional framework for CT: computational concepts, computational practices, and computational perspectives. This framework has been widely applied in teaching practices to enhance students’ CT. Anderson (2016) proposed five core CT competencies: decomposition, pattern recognition, abstraction, algorithmic design, and evaluation. This definition interprets CT as the essential abilities required for problem-solving. Similarly, Shute et al. (2017) developed a CT competency model comprising six dimensions: decomposition, abstraction, algorithms, debugging, iteration, and generalization.

While definitions of CT are diverse—such as Wing’s macro-level definition, Brennan and Resnick’s practical framework, and Shute et al.’s process model—their core consistently points to a set of decomposable and measurable cognitive skills. Therefore, to accurately assess the differential impact of Generative AI on CT sub-skills, this study deconstructs and reintegrates CT competencies based on Anderson’s core CT abilities and Shute et al.’s CT competency model, forming the core CT competency framework for this research: Algorithmic and Modeling, Pattern Recognition and Evaluation, and Abstraction and Decomposition.

The three dimensions of this framework are defined as follows:

Algorithmic and modeling refers to the ability to design and analyze ordered sequences of instructions to solve problems and to model systems, emphasizing the logical transition from understanding a problem to creating a solution (Shute et al., 2017).

Pattern recognition and evaluation refers to the ability to identify inherent patterns, regularities, or similarities within data, problems, or systems, and to judge the effectiveness, strengths, and weaknesses of solutions, models, or algorithms (Sung, 2022; Wu and Chen, 2022).

Abstraction and decomposition refers to the ability to strip away details to grasp the core essence of a problem and to break down a complex whole into manageable parts, serving as the starting point for tackling complex problems (Boom et al., 2022; Shute et al., 2017).

This framework encompasses the core elements of CT [such as decomposition, abstraction, algorithms, and evaluation emphasized by Anderson (2016)]. Consequently, this study utilizes this framework to design instructional strategies and assess the development of students’ computational thinking.

### 2.4 Research review and gap

Research indicates that utilizing Generative AI to assist in programming can significantly enhance students’ computational thinking and programming abilities. In a programming course, Yilmaz and Karaoglan Yilmaz (2023a) required students to use ChatGPT for programming exercises. After a 5-week instructional period, they found that undergraduate students’ computational thinking skills, programming self-efficacy, and class engagement were significantly higher than those of the control group. Liao et al. (2024) designed an intelligent programming scaffolding system based on ChatGPT and conducted a mixed-methods study. The results showed that most undergraduate students held a positive attitude toward the system, and it effectively improved their computational thinking.

Through a review of existing literature, we find that while the potential advantages of Generative AI in programming education have begun to emerge, research in this area remains in its early stages and has primarily focused on higher education settings (Nathaniel et al., 2025). However, high school students are at a critical period of cognitive development, and their cognitive characteristics differ significantly from those of undergraduate and postgraduate students. Consequently, the cultivation of their computational thinking differ from those of university students. Currently, within K-12 education, there is a notable research gap concerning the integration of Generative AI with programming education to foster students’ higher-order thinking, with relatively few related studies published. Furthermore, most existing research measures computational thinking as a holistic construct, lacking a fine-grained analysis of its impact on sub-dimensions such as abstraction and decomposition, algorithmic and modeling, and pattern recognition. This approach obscures the potential differential effects of Generative AI on specific CT components. Based on this, the present study proposes the following research questions:

1. Compared to traditional teacher-led instruction, what is the impact of Generative AI-assisted programming on high school students’ computational thinking (abstraction and decomposition, algorithmic modeling, pattern recognition and evaluation)?

2. What are high school students’ perceptions of generative artificial intelligence and its use in programming instruction?

## 3 Methodology

### 3.1 Participants

This study was conducted in a high school located in Western China. The tenth grade at this school consisted of eight classes, all of which were parallel classes (i.e., academically similar). As the research was carried out in an authentic educational setting, it was not feasible to disrupt the original class compositions to randomly assign students to experimental and control groups. Therefore, from the eight parallel classes, one class was randomly selected to serve as the experimental group, while another class was designated as the control group. The study involved 83 tenth-grade students with an average age of 15. The experimental group (*n* = 42) consisted of 23 boys and 19 girls, while the control group (*n* = 41) consisted of 22 boys and 19 girls. Almost all these students had little exposure to Python text-based programming before the intervention. The results of the independent-samples *t*-test showed that there were no significant differences between the two groups of students in terms of computational thinking before the intervention (*p* > 0.05).

It is important to note that this experiment was conducted in an authentic classroom setting using a quasi-experimental design, without random assignment of participants, which may introduce selection bias. Although we randomly selected intact classes to ensure no significant differences in baseline levels between groups, potential differences between the students in the two groups may still exist. Therefore, this factor should be considered when interpreting the results.

Furthermore, since all participants were from the same school, the external validity of the study’s conclusions is somewhat limited. Caution is therefore warranted when generalizing the findings to different regions or school contexts.

### 3.2 Ethical considerations

This study was conducted as a teaching practice research within the regular educational environment in China, aiming to explore effective instructional strategies. The research design strictly adhered to China’s Law of the People’s Republic of China on the Protection of Minors, Personal Information Protection Law of the People’s Republic of China, and relevant guidelines for educational research ethics issued by the Ministry of Education to safeguard the rights and welfare of the participating students.

As an innovative practice initiative of the school, this study received review and approval from the school’s Academic Affairs Office. The implementation process was carried out under the supervision of both the Academic Affairs Office and the Teaching and Research Group.

Since all participants were minors, we rigorously followed the following ethical protocols: Prior to the start of the study, we distributed an Informed Consent Form to both the students’ guardians and the students themselves. The form detailed the research purpose, methods, content, duration, potential benefits and risks, data confidentiality measures, the principle of voluntary participation, and the right to withdraw unconditionally at any time. It was also clarified that non-participation would not affect the student’s grade or standing in the course. The signing rate for the Informed Consent Form was 100%.

Furthermore, all data collected during the experiment were anonymized. Interview recordings were permanently deleted immediately after being transcribed into verbatim transcripts. All data were used solely by the research team for the purposes of this analysis.

### 3.3 Experimental design

This study employed a convergent parallel mixed-methods design. First, during the data collection phase, quantitative data (pre-and post-test questionnaires) and qualitative data (interviews) were collected independently within the same timeframe. Second, statistical analysis was performed on the quantitative data, and thematic analysis was conducted on the qualitative data. Third, the two sets of data were compared, integrated, and triangulated to gain an in-depth understanding of the impact of Generative AI-assisted programming on high school students’ computational thinking.

**FIGURE 1:**
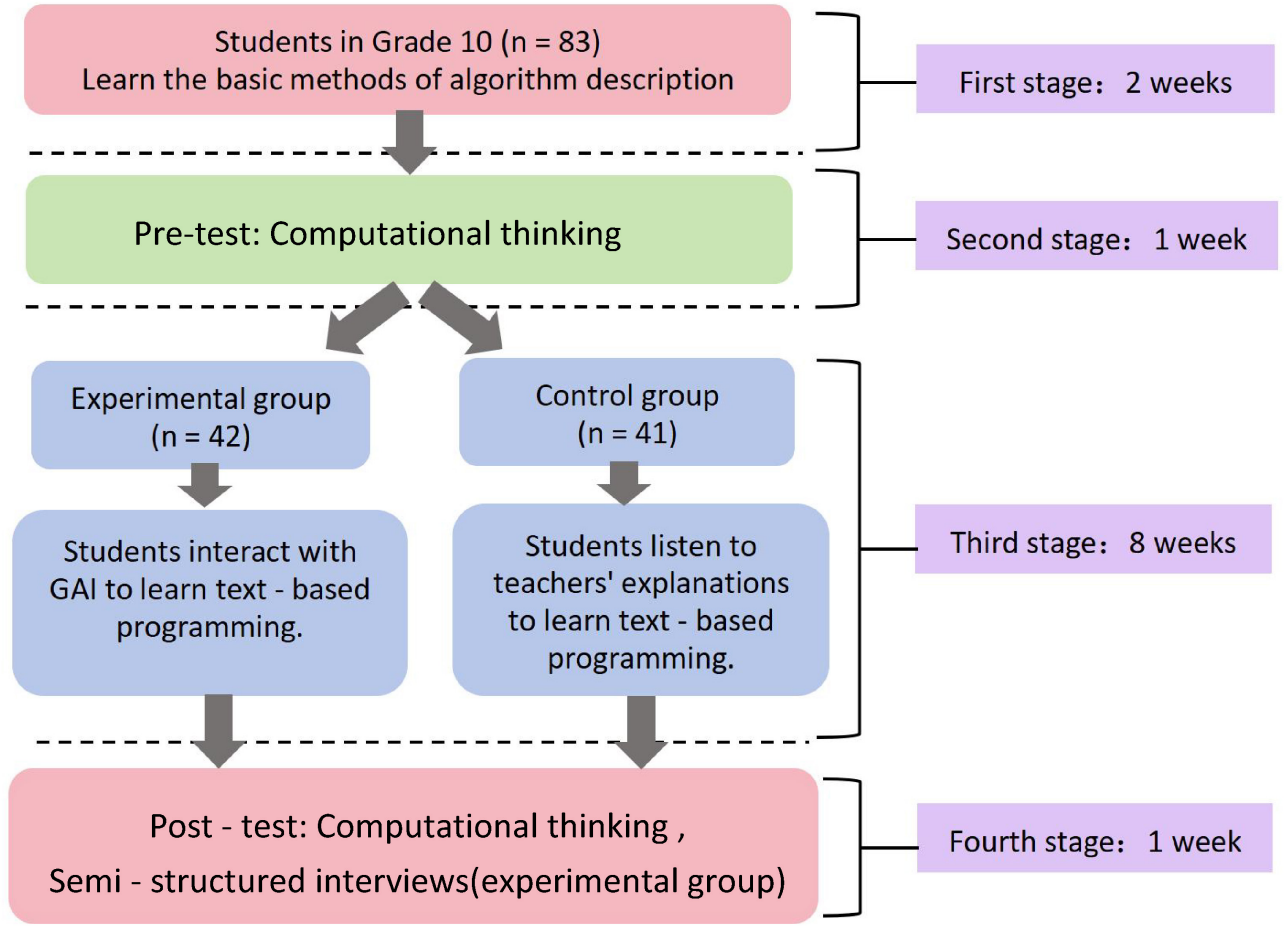
Experimental design.

This study employed a pretest-posttest quasi-experimental design (Figure 1). During the 2 weeks preceding the intervention, all participating high school students received instruction on fundamental algorithmic description methods. In the third week, a pretest was administered to all students to assess their computational thinking.

The teaching intervention was implemented from weeks 4 to 11, consisting of one 45-min session per week. During the intervention, these students utilized Generative AI to learn Python syntax, comprehend code semantics, and debug programs, primarily during the phases of programming debugging and knowledge transfer. In contrast, students in the control group received traditional teacher-led instruction for the corresponding segments, where the teacher explained syntax and code and assisted with debugging.

Before the experiment began, all participants were informed of the study’s purpose and procedures, including a detailed explanation of their roles and tasks—specifically, why the experimental group would use Generative AI during programming activities while the control group would not. After this briefing, consent was obtained from all students for their participation.

Upon conclusion of the intervention, a posttest measuring computational thinking was administered to all students. Subsequently, 18 students from the experimental group were randomly selected to participate in semi-structured interviews, aimed at gaining deeper insights into their perceptions of using Generative AI in learning.

Implementation fidelity control measures. To minimize interference from extraneous variables and ensure the intervention was implemented as planned, the following control measures were enacted.

First, consistency in students’ use of Generative AI was ensured. Students in the experimental group were provided with application strategies (e.g., how to write prompts) and usage guidelines (defining permissible and impermissible uses). Each lesson included a structured task checklist requiring students to complete at least three interactive dialogues with the Generative AI, specifically: (1) inquiring about code meaning, (2) requesting syntax explanations, and (3) debugging code errors. Furthermore, students were required to preserve complete dialogue logs. Researchers periodically reviewed these logs to monitor the content and frequency of interactions.

Second, teacher roles were standardized. To control for teacher variables and ensure consistency in teaching style, the same instructor taught both groups. In the experimental group classes, the teacher’s primary roles were designing learning tasks, maintaining classroom order, and managing pacing. Personalized assistance for programming issues (e.g., code questions, syntax, debugging) was primarily facilitated by guiding students to interact with the Generative AI. In the control group, the teacher directly addressed all code, syntax, and debugging issues through instruction and guidance. To verify adherence to this protocol, a researcher observed and recorded teacher behaviors during each session, analyzing factors such as time spent on direct instruction and patterns of interaction.

Third, instructional content and time were synchronized. Both groups used identical teaching cases, tasks, and covered the same knowledge points. The duration of each lesson and the total number of instructional cycles were also exactly the same for both groups.

Generative AI intervention protocol. Students in the experimental group used a Chinese Generative AI tool (iFlytek Spark, web version). Prior to the course implementation, students in the experimental group received training on how to use the Generative AI, primarily focusing on crafting effective prompts and understanding the scope of permitted use. The intervention lasted for 8 weeks, consisting of one 45-min session per week. During each session, students had approximately 25–30 min to interact with the Generative AI to complete programming tasks. Students interacted under teacher supervision to ensure the use of the Generative AI aligned with the pedagogical objectives.

### 3.4 Measurement tools

#### 3.4.1 Computational thinking

The Bebras CT Challenge was the test tool for computational thinking in this study.^1^ The Bebras CT Challenge is an internationally renowned online competition used to test the computational thinking of students of different age groups (6–18 years old) and has proven to be highly effective (Boom et al., 2022; Dagiene and Jevsikova, 2013; Román-González et al., 2017). The Bebras CT Challenge divides students into six age groups. Since the average age of the students in this experiment was 15 years old, we selected the questions for the 14–16-year-old age group. The questions for each age group are divided into three difficulty levels: A-level (easy), B-level (medium), and C-level (difficult), with different difficulty levels measuring different sub-skills of computational thinking. The A-level consists of 6 questions, each worth 4 points, measuring algorithmic and modeling abilities. The B-level includes 5 questions, each worth 5 points, measuring pattern recognition and evaluation abilities. The C-level contains 5 questions, each worth 6 points, measuring abstraction and decomposition abilities (Sun and Liu, 2024). The full score is 79 points, with a full score of 24 points for the algorithm and modeling dimension, 25 points for the pattern recognition and evaluation dimension, and 30 points for the abstraction and decomposition dimension (Sun and Liu, 2024). These measurement dimensions align with the dimensional framework of this study. In addition, we used SPSS25 to test the validity of the questionnaire. The data results showed that the KR-20 coefficient was greater than 0.700, indicating that the test questions had high reliability. The Cronbach’s alpha coefficient was greater than 0.700, indicating that the test items possessed high reliability.

#### 3.4.2 Semi-structured interviews

Based on the research of Lee et al. (2024), a semi-structured interview outline was adopted to explore the views of students in the experimental group on Generative AI. The interviews mainly posed the following three types of questions to the students: (1) Their overall experience of using Generative AI; (2) The role of Generative AI in the learning of Python text-based programming; (3) Their attitude toward Generative AI in future learning.

#### 3.4.3 Generative AI platform and interaction protocol

This study employed iFlytek’s Spark, a Generative AI platform developed in China. The platform was selected primarily for two reasons: it eliminated language barriers, as students could interact with it in Chinese; and its performance is benchmarked against established models like GPT-4o, with a specific focus on educational applications, making it a widely adopted tool in teaching and learning contexts. The researchers created individual accounts for all participating students to ensure seamless access to the platform throughout the duration of the experiment. To prevent students from becoming overly reliant on the Generative AI tool, we established usage guidelines that clarified both permitted and prohibited scenarios for its use (see Table 2). The core interaction pattern was designed as a student inquiry–AI response loop. Students were instructed to pose their specific difficulties to the Generative AI, carefully comprehend its responses, and engage in further questioning until the problem was resolved.

**TABLE 2:** Guidelines for permitted and prohibited use of Generative AI in programming learning.

Permitted scenarios for Generative AI use (whitelist)	Prohibited scenarios for Generative AI use (blacklist)
When encountering unfamiliar concepts or knowledge gaps.	Prohibited: Requesting code for an entire project.
When encountering syntax errors and not understanding the error message.	Prohibited: Copying and pasting entire code segments without comprehension.
When unable to understand code or having forgotten the usage of specific syntax.	Prohibited: Using Generative AI to complete exercises or tests.
When needing examples to aid understanding of specific syntax or function usage.	Prohibited: Having Generative AI perform algorithmic thinking on your behalf.
After designing an algorithm but being unsure how to implement certain steps in code.	
After completing a draft and needing feedback, suggestions, evaluation, or optimization.	
When stuck on a project and in need of inspiration.	

When using Generative AI, you must lead the entire process with deep cognitive engagement. You must understand all code and suggestions it provides. If generated content is unclear, you should ask follow-up questions or seek help from the teacher or classmates.

#### 3.4.4 Experimental procedure

Four projects were designed for this experiment: Lucky Draw, Intelligent Dietary Advisor, Energetic Breakfast Planner, and Price Guessing Game. Each project incorporated different fundamental grammar knowledge, as detailed in Table 3. The teaching activities were structured around the core components of computational thinking ability. To illustrate the implementation process in detail, the Lucky Draw project is used as an example below.

**TABLE 3:** Projects and the included grammar knowledge.

Project name	Programming grammar knowledge included in the project
Project 1: Lucky Draw	Constants, variables, variable naming rules, common Python data types, data type conversion functions, input and output statements, assignment statements
Project 2: Intelligent Dietary Advisor	Arithmetic operators, relational operators, single-branch structure, double-branch structure, multi-branch structure
Project 3: Energetic Breakfast Planner	for loop structure, range function, usage of continue, usage of break
Project 4: Price Guessing Game	Logical operators, increment and decrement statements, while loop structure

##### 3.4.4.1 Situational interaction

Problem scenarios were created to engage students’ initiative and stimulate their learning interest. This initial phase aimed to establish authentic contexts that motivate students to explore programming concepts.

##### 3.4.4.2 Abstraction and decomposition

During this phase, the teacher worked collaboratively with students to abstract the core problem of the project and decompose the larger problem into smaller, manageable sub-problems. The primary objective of this stage was to cultivate students’ abilities in abstraction and decomposition, which are fundamental to computational thinking.

##### 3.4.4.3 Algorithm design

**FIGURE 2:**
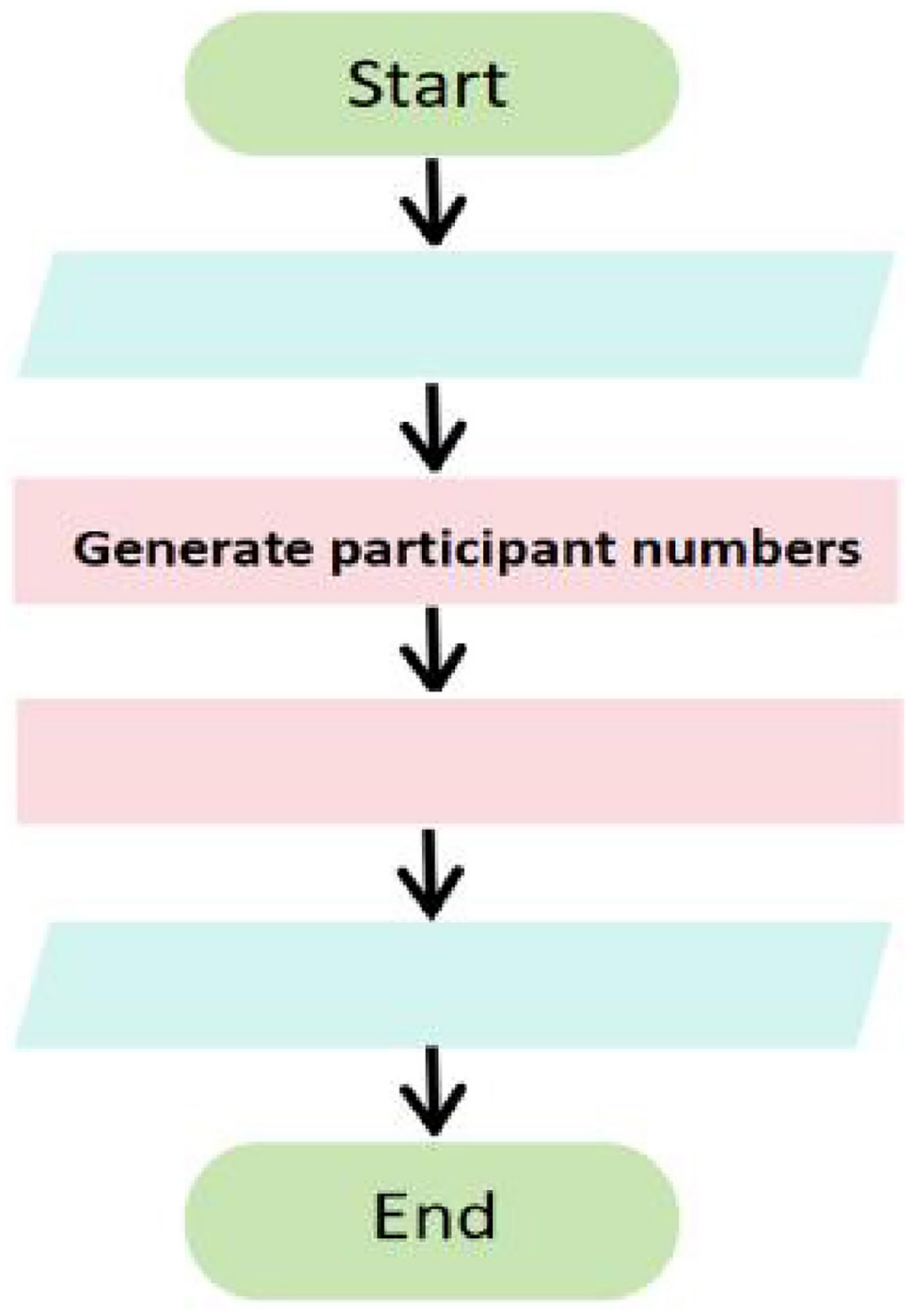
Incomplete flowchart.

Building on their authentic experience and the analysis conducted in the previous step, students attempted to describe the program’s algorithm using flowcharts. This stage was designed to develop students’ rigorous logical thinking. For the Lucky Draw project, the teacher provided an incomplete flowchart (see Figure 2), which students were tasked with completing.

##### 3.4.4.4 Programming and debugging

Given that students had no prior foundation in text-based programming, the teacher presented the complete code to them. Students were tasked with understanding the code’s meaning and mapping it step-by-step to the corresponding sections of the flowchart.

The instructional approach differed between groups during this phase:

Experimental group: Students interacted with Generative AI to learn the code’s meaning. Focusing on code segments they did not understand, students engaged in discussions with the Generative AI. The teacher listed the syntax knowledge students needed to master, after which students inquired about the usage of this syntax and requested explanatory examples from the Generative AI. Students engaged in multiple rounds of dialogue with the Generative AI until they achieved understanding.

Control group: Students learned the code and syntax knowledge through the teacher’s direct explanation. The teacher explained the meaning of the code line by line and provided direct instruction on the included syntax knowledge.

##### 3.4.4.5 Generalization and transfer

The teacher asked students to consider modifying the program’s functionality. Students modified the code, expanded their thinking, and implemented their desired features. For the Lucky Draw project, the teacher required students to modify the random number generator program into a random prize generator. Students referred to the analysis process of the random number generator and, through abstraction, decomposition, and algorithm design, ultimately created their product.

The support mechanisms differed between groups:

Experimental group: Students interacted with the Generative AI to complete the code writing. When students wanted to implement a specific function but lacked the coding knowledge, they sought help from the Generative AI. When encountering runtime errors and struggling to identify the cause, they consulted the Generative AI, requesting analysis of the error and guidance on how to correct it.

Control group: Students modified the code independently. When encountering difficulties, they completed the task with the teacher’s assistance. For code debugging issues, they similarly sought the teacher’s help by raising their hand.

### 3.5 Data collection and analysis

The computational thinking test used in this study was administered and collected online, achieving a 100% valid response rate for the questionnaires. First, a baseline equivalence test was conducted. The results of independent samples *t*-tests on the pre-test scores between the experimental and control groups showed that there were no significant differences in the pre-test scores for overall computational thinking, or its respective sub-dimensions (*p* > 0.05). This indicates that the two groups of students were homogeneous, satisfying the baseline equivalence assumption for the experiment.

To gain a deeper understanding of students’ perceptions of Generative AI and its role in programming, we conducted semi-structured interviews. Based on the post-test results of computational thinking, students were categorized via K-means clustering into low (44–55), medium (56–69), and high (70–79) groups. Then, the researchers randomly selected 6 students from each of the three score ranges (high, medium, and low), totaling 18 students as interview subjects. The analysis of interview data followed four steps: recording the interviews, reading the interview transcripts, coding the data, and interpreting the data (Ruona, 2005). This study employed content analysis techniques to identify codes, themes, and their frequencies in the qualitative data. The analysis began with the transcription of interview recordings and an initial round of coding. This process led to the development of a structured coding framework comprising three main themes (students’ perceptions of Generative AI, its role in programming, and future usage intentions) and 18 sub-themes. To ensure the reliability and objectivity of the coding process, a second, independent researcher was trained on this framework. Following the training, both researchers independently coded a randomly selected subset (30%) of the interview transcripts.

Subsequently, inter-rater reliability was assessed using Cohen’s Kappa coefficient. The analysis resulted in a Kappa value of κ = 0.70, indicating a substantial level of agreement between the coders. Any discrepancies in the initial coding were resolved through discussions between the two researchers, which served to clarify the definitions of the sub-themes and refine the coding framework. Following this consensus-building and framework refinement, the first author proceeded to code the entirety of the remaining transcripts.

## 4 Results

In this study, repeated-measures analysis of variance was used to investigate the impact of the Generative AI-assisted learning strategy on students’ computational thinking over time (pre-test and post-test). The Generative AI-assisted strategy was a between-subjects factor, and time was a within-subjects factor. The main effects, interaction effects, and simple effects of the analysis of variance were set at *p* < 0.05. The Mauchly’s test of sphericity was used to examine the validity of this assumption. The test result for the sub-dimension was statistically non-significant, meeting the assumption of sphericity (*p* = 1.000). Next, we will analyze and explain the results one by one according to the three research questions proposed at the beginning of the study.

Question 1: What is the impact of Generative AI on high-school students’ computational thinking (abstraction and decomposition, algorithmic modeling, pattern recognition and evaluation)?

Table 4 presents the results of the repeated-measures ANOVA for computational thinking. A repeated-measures ANOVA revealed a significant interaction between teaching method and testing time for overall computational thinking, *F*(1, 81) = 14.29, *p* < 0.001, ηp^2^ = 0.150. According to Cohen’s (1988) guidelines (where partial η^2^ values of 0.01, 0.06, and 0.14 correspond to small, medium, and large effect sizes, respectively), this partial η^2^ value indicates a large effect size. In educational practice, a large effect size typically indicates that one teaching method can produce substantial, observable differences compared to traditional methods, providing strong evidence of effectiveness for promoting such interventions in similar high school programming courses. To control the Type I error risk associated with testing multiple CT sub-dimensions, the significance level for all subscale analyses was set to α = 0.0125 after applying the Bonferroni correction.

**TABLE 4:** Results of repeated-measures analysis of variance for computational thinking.

Dimension	Group	N	Pre-test M (SD)	Post-test M (SD)	Pre-post difference (95% CI)	ANOVA		
						Interaction (Method × Time)		
						*F*(df)	*P*	η ^2^
Overall CT skills	EG	42	46.83 (9.65)	59.48 (7.66)	12.65 (10.09, 15.20)	14.286 (1,81)	< 0.001	0.150
	CG	41	46.74 (8.27)	52.48 (9.26)	5.73 (3.14, 8.32)			
Algorithmic modeling	EG	42	17.17 (3.77)	21.38 (3.01)	4.21 (2.97, 5.45)	22.315(1,81)	< 0.001	0.216
	CG	41	17.78 (3.88)	17.80 (4.58)	0.02 (−1.23, 1.28)			
Pattern recognition and evaluation	EG	42	15.62 (4.32)	15.36 (4.33)	−0.26 (−1.40, 0.87)	5.194 (1,81)	0.025	0.060
	CG	41	15.00 (4.18)	16.59 (3.94)	1.59 (0.44, 2.73)			
Abstraction and decomposition	EG	42	13.76 (6.83)	22.43 (4.89)	8.67 (6.81, 10.53)	11.065 (1,81)	0.001	0.120
	CG	41	13.89 (5.37)	18.13 (5.16)	4.24 (2.36, 6.13)			

EG, Experimental Group; CG, Control Group; CI, Confidence Interval. η^2^ values are partial eta squared. The significance level for subscale analyses was adjusted to α = 0.0125 using the Bonferroni correction.

Regarding the sub-dimensions, the interaction between teaching method and time was highly significant for Algorithmic Modeling, *F*(1, 81) = 22.32, *p* < 0.001, ηp^2^ = 0.216 (large effect), and significant for Abstraction and Decomposition, *F*(1, 81) = 11.07, *p* = 0.001, ηp^2^ = 0.120 (medium effect).

**FIGURE 3:**
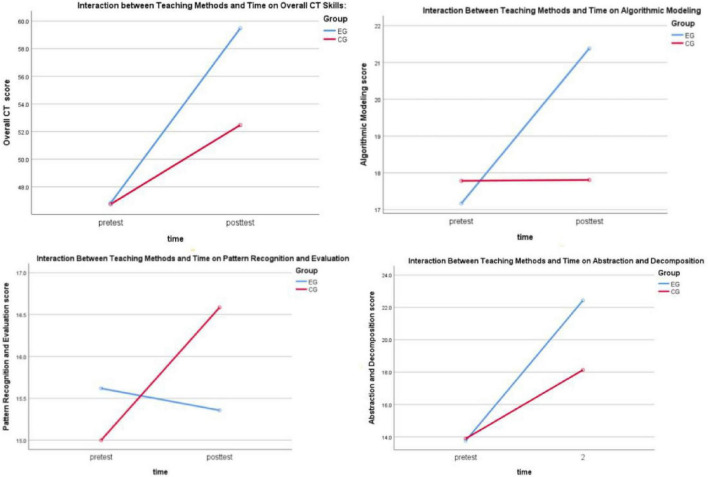
Between-group differences in overall computational thinking and its sub-dimensions.

Simple effects analysis for overall CT skills (with all pairwise comparisons Bonferroni-adjusted) showed no significant difference between the two groups at pre-test (*p* > 0.05). However, at post-test, students in the Generative AI-assisted group scored significantly higher than those in the teacher-led group (*p* < 0.001; see Figure 3). At post-test, the experimental group also scored significantly higher than the control group on both aforementioned sub-dimensions (all *p* ≤ 0.001).

More importantly, in terms of gain scores, the improvement in the total computational thinking score for the experimental group [*M* = 12.65, 95% CI (10.09, 15.20)] was both statistically and substantially greater than that of the control group [*M* = 5.73, 95% CI (3.14, 8.32)], with no overlap in the confidence intervals of the improvement magnitudes for the two groups.

For the Algorithmic Modeling dimension, the experimental group showed a significant gain scores [*M* = 4.21, 95% CI (2.97, 5.45)], while the control group’s gains was minimal and non-significant [*M* = 0.02, 95% CI (−1.23, 1.28)]. The confidence intervals for the improvements of the two groups were completely separate, showing no overlap.

In the Abstraction and Decomposition dimension, the experimental group showed significant gains [*M* = 8.67, 95% CI (6.81, 10.53)], and the control group also gains significantly [*M* = 4.24, 95% CI (2.36, 6.13)]. The confidence intervals did not overlap, with the lower bound of the experimental group’s improvement (6.81) exceeding the upper bound of the control group’s improvement (6.13). These results confirm that Generative AI-assisted programming was more effective than the teacher-led instruction method in promoting the development of these specific CT skills.

However, for the Pattern Recognition and Evaluation dimension, the interaction between teaching method and time did not reach significance after Bonferroni correction [*F*(1, 81) = 5.19, *p* = 0.025, ηp^2^ = 0.060; corrected α = 0.0125]. The within-group comparison results were more complex: the experimental group showed no significant gains [*M* = -0.26, 95% CI (-1.40, 0.87)], while the control group showed a significant gains [*M* = 1.59, 95% CI (0.44, 2.73)]. The partial overlap of the confidence intervals aligns with the non-significant interaction effect (after correction), indicating that Generative AI-assisted learning did not produce superior effects compared to traditional instruction in this dimension. This result indicates that, within the context of this study, Generative AI-assisted learning did not demonstrate the expected facilitative effect on the specific competency of “Pattern Recognition and Evaluation”; its performance even showed a certain contrast with that of traditional instruction.

Overall, the impact of Generative AI-assisted programming on the sub-dimensions of computational thinking exhibited a differentiated pattern. Its intervention effect was most prominent in the Algorithmic and Modeling dimension (ηp^2^ = 0.216, large effect) and also showed a moderately significant effect in the Abstraction and Decomposition dimension (ηp^2^ = 0.120, medium effect). These results align with findings from existing research (e.g., Zhao et al., 2025). However, no significant advantage of Generative AI over traditional instruction was found in the Pattern Recognition and Evaluation dimension (the interaction effect was non-significant after correction); in fact, the control group showed some improvement in this dimension. The potential reasons and pedagogical implications of this unexpected specific finding will be explored in-depth in the Discussion section in conjunction with power analysis.

Question 2: What are high-school students’ views on Generative AI and its use in programming teaching?

Students’ perceptions of Generative AI demonstrated positive recognition, but also included reflective consideration of its limitations (key viewpoints are summarized in Table 5).

**TABLE 5:** High-school students’ views on Generative AI.

Sub-themes	Frequency	Example phrases
Advantages		
Personalized learning	15	Generative AI can take care of every student. If I don’t understand something, I can ask AI. But teachers have to take care of many students in the class, which is quite troublesome. Different students may have different questions.
Easy to use	6	Actually, I got used to this software very quickly because its operation is relatively simple.
Timely response	5	Its advantage is that you can interact with it about all your questions, and it can answer very quickly, answering you in a timely manner.
Detailed answers	5	The way AI teaches is more vivid. Different ways of asking questions can also give me different insights. It explains things very meticulously.
Enhanced initiative	4	I think being able to communicate with AI has enhanced my initiative.
Disadvantages		
Unsatisfactory answers	9	Sometimes its language is too difficult to understand. The answers may be beyond your comprehension.
Dependency	3	Maybe I’ll rely on AI. Sometimes, for example, if I don’t understand just one point, I may not think much and directly rely on AI to tell me the answer, thus losing my own thinking.

The value of personalized learning. Students generally believed that Generative AI provided one-on-one tutoring that is difficult to achieve in traditional classrooms, allowing programming learning to proceed at their own pace. For instance, student S15 explicitly pointed out its complementary value: “After all, in class, the teacher is addressing many students, and it’s impossible for me alone to have the teacher tutor me one-on-one. So having an AI that can focus more on my own situation…” Another student, S6, described how AI helped digest knowledge: “During programming, the teacher covers so much in one lesson, I always forget. Then I ask the AI while following along…Although its answers differ from the teacher’s, compared to the teacher’s long explanations, what the AI says allows me to read and understand it slowly, bit by bit.” These experiences align with Vygotsky’s Zone of Proximal Development theory, suggesting that Generative AI acted as a dynamic scaffold, helping students construct understanding at their individual cognitive levels. This corroborates the quantitative findings showing improvement in dimensions such as abstraction/decomposition.

Ease of use and rapid responsiveness. Students reported that the simplicity of use and immediate responsiveness of Generative AI made the learning process more proactive. For example, student S1 said, “I actually adapted to this software very quickly because, well, its operation is relatively simple.” Thirteen percent of students felt the response speed of Generative AI was very fast, allowing them to ask questions anytime, anywhere. They mentioned, “It’s also more efficient, able to answer quickly, perhaps faster and more prompt than a teacher,” and “It also sped up our learning.” This result indicates that the low barrier to entry and rapid response characteristics of Generative AI significantly reduced students’ sense of intimidation toward programming, greatly increasing their willingness to ask questions and forming a positive cycle of active inquiry → receiving feedback. This positive learning experience made the process more self-directed.

Reflection on technological dependency and answer quality. While acknowledging the advantages, some students also expressed concerns, most notably about the risk of over-reliance on AI for thinking. For instance, student S11 mentioned in the interview: “It’s possible that I might become dependent on AI. Sometimes, for example, if I don’t understand just this one point, I might not think too much about it and would directly rely on AI, use AI, and let AI tell me, essentially losing my own thinking.” This suggests Generative AI may pose a potential risk of “cognitive substitution,” leading to intellectual passivity, where students seek answers directly from Generative AI without engaging in thought, which could hinder the development of their critical thinking. This finding provides evidence to help explain the lack of significant change in the Pattern Recognition and Evaluation dimension in the quantitative results.

Furthermore, approximately nine students noted that Generative AI’s answers were “not very easy to understand” (S14). This implies students need to engage in further processing of the AI’s content. Dissatisfaction with the quality of Generative AI’s responses could potentially reduce learning enthusiasm, interrupt the learning process, or cause frustration. This imprecise feedback may make it difficult for students to categorize and evaluate coding solutions, which could also explain the lack of significant change observed in the quantitative results for the Pattern Recognition and Evaluation dimension.

### 4.1 The cognitive role of generative AI in text-based programming learning

Students provided specific descriptions of how Generative AI assisted their programming practice at different levels (see Table 6). These descriptions clearly map onto its mechanisms for reducing cognitive load and supporting higher-order thinking.

**TABLE 6:** The role of Generative AI in text-based programming.

Sub-themes	Frequency	Example phrases
Code explanation	13	The main use of AI is to help understand the code. That is, if I don’t understand a certain line, I can ask it to explain.
Learning grammar knowledge	5	In terms of programming, I think it can teach us some programming knowledge and is more convenient.
Giving examples	5	AI will give some examples. For instance, it will give examples about things like strings that we learned before. I think it makes things clearer.
Improving programming efficiency	4	Sometimes, when I don’t know how to implement some programming segments or functions, I can directly ask AI. Then I can assemble the code myself, which can speed up my programming. Also, I can think about innovating on my own.
Code writing	3	It can help me write the code directly.
Correcting code errors	2	I can hand the code I wrote to AI and let it show me what mistakes I’ve made.
Providing ideas	2	Sometimes when I get stuck in my thinking or don’t know how to solve a problem, it can also provide a solution.

#### 4.1.1 Code explanation

Over one-third of the students (38%) discussed using AI to explain code. For example, student S17 stated: “I mainly use AI to have it help me understand the code, meaning if there’s a line I don’t understand, I ask it to explain it.” This essentially transforms abstract code symbols into comprehensible natural language, helping students overcome comprehension barriers. Here, Generative AI serves a role in cognitive offloading, providing process-based evidence for the improvement in the Abstraction dimension observed in the quantitative results.

#### 4.1.2 Syntax support and efficiency enhancement

Students utilized Generative AI to recall syntax knowledge (14.7%) and correct code errors. This directly reduced the cognitive resources consumed by syntactic details and debugging. The described use of AI for learning and recalling syntax knowledge functioned to lower extraneous cognitive load, allowing them to focus on algorithmic logic. As student S9 noted: “I can give the code I wrote to AI and have AI check what errors I have.” Furthermore, students mentioned that after freeing up cognitive resources, they had more energy for “innovation.”

Finally, we inquired about the likelihood of students using Generative AI in their future studies and for what purposes. Their views are summarized in Table 7. When asked if they would use Generative AI in the future, only one student indicated they would not, while the vast majority expressed their intention to continue using it. Many students (44%) primarily viewed Generative AI as a Q&A tool, a superior alternative to search engines. As S17 stated, “When encountering unfamiliar concepts, I ask AI directly, having it explain using knowledge learned at this stage.” S7 said, “You can ask it various questions.” Some students also mentioned that Generative AI could provide them with more inspiration (16.7%) and assist them in solving problems (16.7%). S15 noted, “I usually use the AI you set up for us at home to provide me with ideas and such; I use it for all sorts of things.” A small number of students mentioned they would use Generative AI for writing and foreign language learning.

**TABLE 7:** The usage of Generative AI in future learning.

Sub-themes	Frequency	Example phrases
Answering various questions	8	Of course I’ll use AI. It’s really convenient. Its usage is simple, and you can ask it all kinds of questions.
Providing inspiration	3	In the future, when writing essays or making PPTs, if I have no inspiration, I’ll ask AI to come up with ideas on what to do. Then I’ll complete my essays, hand-written newspapers, etc. based on its suggestions. It provides some inspiration.
Assisting in solving problems	3	If there are some problems that are very difficult for you to solve on your own, you can use AI to help solve them.
Aiding in writing	2	In the future, when I don’t know how to write an essay, I can also ask it.
Learning foreign languages	1	There’s a high probability of using it in daily life. When I’m translating documents, I’ll give it the English text and ask it to tell me the Chinese or other languages.
Not using	1	If it’s for daily life, it doesn’t seem to be very useful. I may not use it in the future.

Across all interview results, no student mentioned that Generative AI could help them “summarize code” or “compare the pros and cons of different solutions,” or similar higher-order analytical tasks. This observation further corroborates the finding of no significant improvement in the Pattern Recognition and Evaluation dimension.

## 5 Discussion

### 5.1 Support for and extension of existing research

The primary aim of this study was to investigate the impact of Generative AI on the computational thinking of high school students within the context of Python text-based programming. The results indicate that, compared to traditional lecture-based instruction, Generative AI-assisted programming can significantly enhance the computational thinking of high school students. This finding supports and extends similar conclusions drawn from research in higher education settings (Yilmaz and Karaoglan Yilmaz, 2023b; Zhao et al., 2025), suggesting that the effectiveness of Generative AI as a cognitive tool can be extended to the K-12 stage.

A fine-grained analysis of the sub-dimensions revealed a dimension-specificity in the effects of Generative AI. Specifically, students’ abilities in algorithmic modeling and abstraction/decomposition showed significant improvement, which aligns with the findings of Liao et al. (2024). However, no significant changes were observed in students’ pattern recognition ability.

This observed dimension-specificity suggests that the positive effects of Generative AI are primarily concentrated in cognitive processes that are naturally well-matched to its technical affordances, such as handling structured logic and providing immediate feedback. For higher-order thinking skills that require active induction, evaluation, and questioning, without targeted instructional design, Generative AI may yield minimal benefits or even pose a risk of cognitive substitution.

### 5.2 Interpretation of the reasons for Generative AI’s differential impact on computational thinking

To explain the differential impact of Generative AI, we interpret it from the perspectives of technological affordance, cognitive processes, and theoretical support. Regarding the two dimensions that showed significant improvement—algorithmic modeling ability and abstraction/decomposition ability—Generative AI played the role of cognitive offloader. For programming novices, when solving complex programming problems, their working memory is easily overwhelmed by trivial details such as syntax and error codes. This heavy extraneous cognitive load occupies substantial limited working memory capacity, leaving insufficient germane cognitive load for higher-order thinking like algorithmic design. Generative AI took over tasks such as locating errors and explaining code (as noted by student S17: “If there’s a sentence the student doesn’t understand, just ask it to explain”), thereby reducing the cognitive load induced by syntactic details. This allowed students to reallocate the freed cognitive resources to abstraction/decomposition, algorithmic design, and modeling. Concurrently, they could engage in more creative activities. As student S19 mentioned, using Generative AI for programming improved efficiency, freeing up time to think about how to combine code and innovate programs. Generative AI can help students translate ideas into code, serving to externalize implicit thinking. This facilitates the transition from abstract thinking to concrete modeling, which in turn reinforces students’ abstract and decompositional thinking, greatly strengthening the formation of abstraction/decomposition abilities.

The ICAP framework posits that the most effective mode of cognitive engagement is interactive (Chi and Wylie, 2014). Students engaged in multiple rounds of in-depth dialogue with Generative AI to solve problems. This mode aligns with the characteristics of interactive activities within the ICAP framework, which are believed to induce the deepest level of cognitive processing, thereby fostering the development of higher-order thinking. This also explains why the computational thinking of the experimental group was significantly superior to that of the control group.

The experimental results indicated that students’ abilities in pattern recognition and evaluation did not show significant improvement. We analyze the reasons for this from a cognitive perspective. Pattern recognition and evaluation rely on learners’ active observation, induction, and comparison. These aspects currently remain strengths of human teachers. Teachers can guide students through more refined pattern discrimination and evaluation exercises by asking questions, providing examples, and making comparisons. Consequently, students in the control group showed significant improvement in this dimension. In contrast, students in the experimental group used Generative AI to assist their programming. The powerful generative capability of Generative AI can easily lead students to directly produce the desired content, bypassing the cognitive process of active exploration (as student S11 admitted, “I couldn’t be bothered to observe”). This likely explains the lack of significant change observed in this group.

### 5.3 Potential influencing factors of the research findings

While interpreting the aforementioned mechanisms, it is crucial to carefully consider the potential confounding variables not controlled for in this study. They provide an important perspective for understanding the precise boundaries of our findings.

First, differences in students’ prior experience may have moderated the intervention outcomes. The study did not systematically measure students’ pre-existing metacognitive skill levels. It can be speculated that students with richer prior knowledge and higher metacognitive levels could collaborate with Generative AI more efficiently to solve problems, engage in deeper interactions, and exercise critical questioning. In contrast, absolute novices or students with lower metacognitive levels likely depended more heavily on the content provided by Generative AI and accepted it uncritically, thereby benefiting less in areas like pattern recognition. This may partially explain the overall lack of significant change in that dimension.

Second, the “process black box” of Generative AI use limits the explanation of micro-level mechanisms. The research process relied on pre/post-tests and retrospective interviews, failing to capture precise data on the frequency and content (e.g., prompts) of student-AI interactions through log data. For instance, the lack of process data makes it impossible to determine whether the lack of improvement in critical thinking stems from students’ uncritical acceptance of outputs or from deficiencies in their questioning techniques.

Third, constraints related to ecological validity and the measurement tools themselves. The intervention was conducted in a relatively controlled instructional environment, which differs from scenarios where students use Generative AI completely autonomously.

Fourth, the duration and depth of the intervention. The 2-month experiment might have been insufficient for significant changes to occur in abilities like pattern recognition, which require long-term immersion. In terms of activity design, the primary use of Generative AI in this study’s programming activities was for interactive learning to address students’ syntactic and coding problems. No activities were specifically designed to target pattern recognition skills. Consequently, the entire instructional process did not trigger cognitive processes such as induction, summarization, reflection, or questioning, which is likely a major reason for the lack of observed change in abilities like pattern recognition.

### 5.4 Implications and recommendations for programming instruction

To maximize the value of Generative AI in programming instruction, this study proposes the following specific teaching strategies.

Design targeted metacognitive scaffolds. To develop skills such as pattern recognition, teachers should design tasks that require comparison, synthesis, and reflection. For instance, during algorithm design, ask students to compare the advantages and disadvantages of different approaches. When interacting with Generative AI, prompt them to synthesize insights by succinctly summarizing its outputs. Furthermore, after Generative AI proposes a solution, students should extract its underlying logic and evaluate it based on criteria like efficiency, readability, and functionality. Moreover, the optimal pedagogical approach may not involve choosing between human teachers and Generative AI assistance, but rather leveraging their complementary strengths. Teachers can design blended instructional models that foster human-AI collaboration, harnessing the respective advantages of each.

Implement training in critical prompt engineering. The quality of student questions shapes their interaction with Generative AI and, ultimately, their learning. Therefore, prompt-crafting should be integrated into instructional goals. Teachers should steer students away from simply asking Generative AI for answers and toward crafting prompts that foster critical thinking—for example, “Why is this code inefficient? Analyze the causes and suggest two optimizations.” Additionally, rather than allowing students to generate entire code blocks, teachers should guide them through an iterative process of step-by-step refinement. Students might write a basic code version, then incrementally add complexity and functionality, turning to Generative AI for targeted assistance only when necessary.

## 6 Conclusion and limitations

### 6.1 Research conclusion

This study aimed to investigate the impact of Generative AI-assisted programming on the computational thinking of high school students. Employing a convergent parallel mixed-methods design, a quasi-experimental study was conducted with 83 tenth-grade students. The main conclusions are as follows:

Impact on computational thinking: The study demonstrated a differential impact of Generative AI-assisted programming within our instructional framework. While it led to marked gains in core competencies like algorithmic thinking and abstraction/decomposition, it failed to enhance abilities in pattern recognition and evaluation over the intervention period.

Qualitative findings: Student interviews underscored a strong appreciation for Generative AI’s timely, personalized feedback and its perceived utility in learning. However, outcomes were often constrained by prompt quality, and a concerning trend of “cognitive substitution”—where students bypassed deep thinking—was noted.

In summary, the evidence provided by this study indicates that in high school Python programming learning, Generative AI, serving as a cognitive tool, can targetedly enhance specific higher-order thinking skills. However, its effects are not universal; rather, they are highly dependent on targeted instructional activities aligned with specific learning objectives.

### 6.2 Limitations

This study has several limitations. First is the sample. This experiment adopted a quasi-experimental design, and all participants were from a single middle school in western China (*N* = 83). While this sampling method ensured feasibility of implementation, it raises concerns regarding ecological validity and limits the external validity of the research conclusions. The sample exhibited high homogeneity and did not involve random assignment. Therefore, the generalizability of our findings to student populations with different cultural backgrounds, educational resources, or grade levels should be treated with caution.

Second is the duration. Limited by the available class hours, this study lasted for 8 weeks, which is a relatively short period. Although improvement in core computational thinking competencies was observed, the development of higher-order abilities often requires long-term accumulation and repeated practice. The current intervention may have only initiated preliminary changes in these skills, without capturing their stable progression.

Third, there are limitations concerning intervention process control and measurement tools. The study did not employ technical means to collect detailed log data of student interactions with the Generative AI, such as interaction frequency and content. Consequently, the understanding of the key mechanism of “how to use Generative AI most effectively” remains a “black box.”

Fourth, there exist differences in the algorithms and training datasets among different Generative AI platforms, leading to variations in their generated content. In practice, educators need to select the most suitable platform based on subject characteristics, student profiles, and the learning environment. The rapid evolution of AI technology is another factor; the specific platform used in this study has undergone continuous upgrades, with improvements in performance and stability, since the quasi-experiment concluded. Therefore, it is necessary to continuously track the impact of Generative AI on teaching in the future. Additionally, despite our efforts to monitor usage through log sampling, individual differences in student-Generative AI interactions persisted, primarily manifested in prompting strategies and the assimilation of feedback. This introduced potential noise regarding implementation fidelity. Hence, future research should aim to design systematic prompting strategy frameworks and interaction quality analysis schemes to enhance the quality of interventions.

### 6.3 Recommendations for future research

Synthesizing the above conclusions and limitations, future research could be conducted in the following areas.

Deepening the investigation into underlying mechanisms. Apply learning analytics to collect and analyze process data from student-Generative AI interactions, uncovering links between specific interaction patterns and learning outcomes.

Optimizing intervention design. Design tasks that incorporate comparison, summarization, and reflection specifically targeting areas like pattern recognition and critical thinking. Optimize teaching strategies to address the dimensions that showed no improvement in this study.

Expanding the scope and depth of research. To strengthen generalizability, future work should employ robust, multi-site RCTs (randomized controlled trials) with larger, more diverse populations, treating factors like prior knowledge as key moderators. Furthermore, longitudinal designs are needed to track the sustained and developmental impact of Generative AI on learning over extended periods.

## Data Availability

The raw data supporting the conclusions of this article will be made available by the authors, without undue reservation.

## Footnotes

1
https://www.bebras.uk
